# Body composition parameters and sarcopenia in adults with Down syndrome: a case–control study

**DOI:** 10.1007/s40520-023-02680-9

**Published:** 2024-03-29

**Authors:** Emanuele Rocco Villani, Graziano Onder, Emanuele Marzetti, Helio Coelho-Junior, Riccardo Calvani, Antonella Di Paola, Angelo Carfì

**Affiliations:** 1 Department of Geriatrics, Orthopedics and Rheumatology, Università Cattolica del Sacro Cuore, Rome, Italy; 2 Università Cattolica del Sacro Cuore, Rome, Italy; 3 Fondazione Policlinico Universitario “Agostino Gemelli” IRCCS, Rome, Italy

**Keywords:** Down syndrome, DXA scan, Sarcopenia, Sarcopenic obesity, Fat mass index, Premature aging

## Abstract

**Background:**

Individuals with Down syndrome (DS) experience premature aging. Whether accelerated aging involves changes in body composition parameters and is associated with early development of sarcopenia is unclear.

**Aims:**

To compare parameters of body composition and the prevalence of sarcopenia between adults with DS and the general population.

**Methods:**

Body composition was assessed by whole-body dual-energy X-ray absorptiometry (DXA). Fat mass (FMI) and skeletal mass indices (SMI) were calculated as the ratio between total body fat mass and appendicular lean mass and the square of height, respectively. Fat mass distribution was assessed by the android/gynoid fat ratio (A/G). Sarcopenia was defined according to the criteria and cut-points recommended by the European Working Group on Sarcopenia in Older People 2 (EWGSOP2). Data on age- and sex-matched non-DS controls were retrieved from the 2001–2002 National Health and Nutrition Examination Survey (NHANES) population.

**Results:**

Sixty-four DS adults (mean age 37.2 ± 12.0 years, 20.3% women) were enrolled and compared with age- and sex-matched NHANES participants (n = 256), in a 1:4 ratio. FMI (7.96 ± 3.18 kg/m^2^ vs. 8.92 ± 4.83 kg/m^2^, *p* = 0.135), SMI (7.38 ± 1.01 kg/m^2^ vs. 7.46 ± 2.77 kg/m^2^, *p* = 0.825) and A/G (0.98 ± 0.17 vs. 1.01 ± 0.22, *p* = 0.115) were not significantly different between DS and control participants. When the sample was stratified by sex, women with DS had a higher FMI compared with their NHANES controls (10.16 ± 4.35 kg/m^2^ vs. 8.11 ± 4.29 kg/m^2^, *p* < 0.001), while men with DS had lower A/G ratio (1.04 ± 0.16 vs. 1.11 ± 0.22, *p* = 0.002). Sarcopenia was more frequent in individuals with DS than in controls (35.6% vs. 19.9%, *p* = 0.007). This association was stronger in men 40 years and older.

**Conclusions:**

Adults with DS have a higher prevalence of sarcopenia compared with the general population. This finding suggests that DS is associated with early muscle aging and calls for the design of interventions targeting the skeletal muscle to prevent or treat sarcopenia.

## Introduction

Down syndrome (DS) is a genetic disorder characterized by a supernumerary chromosome 21 and is the most common genetic cause of developmental disability and cognitive impairment, with an estimated incidence of 1/600 to 1/1000 live births [1]. DS was classified in 1978 by George Martin as a “segmental” progeroid syndrome characterized by early, asynchronous aging of various organs and systems, that begins prenatally [2]. As a result, individuals with DS age earlier and faster than those with no DS, which appears more evident for the central nervous system and the musculoskeletal system [3]. Musculoskeletal abnormalities (e.g., low muscle tone, ligament laxity) are typical features of DS and contribute to the development of physical dysfunction [4]. Moreover, a reduced myofiber area has been observed in young Ts65Dn mice (a murine model of DS), suggesting that muscle atrophy may be one of the abnormalities caused by the trisomy of human chromosome 21 [5]. Whether muscle atrophy plays a role in physical function impairment in DS individuals is yet to be established and no cut-points of muscle mass indices specific for individuals with DS are currently available.

Sarcopenia is a multifactorial condition characterized by a progressive and generalized loss of skeletal muscle mass and strength [6]. Its prevalence increases with age [7] and is associated with a greater risk of negative health-related events, including disability, loss of independence, reduced quality of life, and death [8]. Evidence on body composition and sarcopenia-related parameters in individuals with DS in comparison with the general population is elusive. However, there is indication that children and adults with DS have smaller lean mass, greater fat mass, and lower physical performance than non-DS peers [9] [10]. A recent study conducted in 844 adults with intellectual disabilities, also including 111 adults with DS, showed that sarcopenia was an independent risk factor for death during five years of follow-up. However, sarcopenia diagnosis was not based on a direct measure of muscle mass and no comparison with the general adult population was performed [11]. The co-occurrence of sarcopenia and obesity – sarcopenic obesity – involves a greater risk of negative health-related events than either condition alone [12]. Because adults with DS adults show a higher prevalence of obesity than the general population [13], it is conceivable that these individuals might be at greater risk of sarcopenic obesity. However, no study has yet specifically explored this possibility.

To expand the knowledge on the subject, the present study to assess body composition and muscle function parameters in adults with DS and verifying the prevalence of sarcopenia and sarcopenic obesity [12] in comparison with a reference population of age- and sex-matched non-DS controls.

## Methods

This was a cross-sectional observational study with participants recruited among individuals with DS 18 years and older who were consecutively admitted to the geriatric outpatient clinic of the Fondazione Policlinico Universitario A. Gemelli IRCCS (Rome, Italy) from April 2019 to March 2020. Exclusion criteria were unwillingness to provide informed consent, height < 145 cm (to avoid interference with measures of body composition [14]), technical or organizational issues preventing acquisition of dual-energy X-ray absorptiometry (DXA) scans, presence of acute conditions at the time of admission, or presence of conditions involving an estimated life expectancy of < 6 months. The study was conducted in compliance with the principles laid down in the Declaration of Helsinki. The protocol was approved by the Ethics Committee of the Fondazione Policlinico Universitario A. Gemelli IRCCS / Università Cattolica del Sacro Cuore (Rome, Italy). A written informed consent was obtained from all participants or their proxies prior to inclusion.

### Clinical data

A thorough medical history was collected by study physicians. Vitals signs were measured by standardized protocols by trained personnel. A medical graded scale with a stadiometer was used to measure body mass and height. The body mass index (BMI) was calculated as the ratio between body mass (kg) and the square of height (m^2^). A blood sample was collected by venipuncture after overnight fasting and processed for standard blood biochemistry by the centralized hospital laboratory.

### Assessment of body composition parameters

Body composition parameters were measured by whole-body DXA on a Hologic® Discovery A scanner (Hologic, Inc., Bedford, MA). Participants were requested to lie supine on the scanning bed with their arms along the trunk and the palms of their hands in a neutral position. All measurements were obtained by a certified technician. Measured parameters included segmental and total lean and fat mass. The following parameters were derived:

- Gynoid fat mass (recorded in kg). The superior boundary of the gynoid fat mass region was determined by the upper portion of the trochanter major, while the lower boundary was positioned 96 mm below the upper part of the trochanter major. The lateral aspect of this anatomical region was demarcated by the subcutaneous tissue located on the hip [15];
- Android fat mass (recorded in kg). The lower boundary of the adipose tissue region in the android region was determined by the uppermost portion of the pelvis, with the top margin positioned 96 mm above the lower boundary of this region. The lateral aspect of this anatomical region was demarcated by the lateral aspect of the thorax [15];
- The ratio between android fat mass and gynoid fat mass (A/G ratio) [15];
- Fat mass index (FMI, kg/m^2^), defined as the ratio between total body fat and the square of height;
- Appendicular skeletal muscle mass (ASM, kg), calculated from the sum of upper- and lower-limb lean mass;
- Skeletal muscle index (SMI, kg/m^2^), defined as the ratio between ASM and the square of the height.

### Measurement of physical performance

Performance measures included walking speed at usual pace and isometric handgrip strength. Walking speed was measured on a 4-m course at the participant self-selected pace. The fastest of two trials (m/s) was used for the analysis [16]. Isometric handgrip strength was measured using a Jamar handheld hydraulic dynamometer (Patterson Medical Products, Inc., Cincinnati, OH). The test person was seated on a standard chair with the shoulder in a neutral position, the elbow near the trunk and flexed at 90°, and the wrist in a neutral position (thumb up). The contralateral arm remained relaxed under the thigh. Participants performed one familiarization trial and one actual measurement with the dominant hand. The highest reading (in kg) during a 4-s maximal contraction under encouragement was used for the analysis [17]. To account for the potential influence of body size on muscle strength, handgrip strength was normalized by height squared [18].

### Assessment of sarcopenia and sarcopenic obesity

The presence of sarcopenia was established based on the co-occurrence of low muscle mass and dynapenia, using the cut-points recommended by the European Working Group on Sarcopenia in Older 2 (EWGSOP2) [16]. Accordingly, low muscle mass was defined as an SMI ≤ 7.00 kg/m^2^ and ≤ 5.42 kg/m^2^ in men and women, respectively. Dynapenia was operationalized as a handgrip strength < 27 kg in men and < 16 kg in women. Sarcopenic obesity was defined as the simultaneous presence of sarcopenia and a BMI ≥ 30 kg/m^2^ [12].

### Comparison population

Data on control participants were retrieved from the National Health and Nutrition Examination Survey (NHANES) database. NHANES is an ongoing program aimed at assessing health and nutritional status of nationally representative non-institutionalized children and adults across the United States. The 2001–2002 data were used, owing to availability of walking speed and handgrip strength measures [19]. Controls were age- (± 1 year) and sex-matched NHANES participants between 18 and 65 years of age, taller than 145 cm, without missing data for the variables of interest, in a 1:4 ratio with DS participants.

### Statistical analysis

Normality of data was assessed using the Kolmogorov − Smirnov test. Differences in continuous and categorical variables between groups (NHANES vs. DS) were assessed by independent samples Student’s t-test and χ^2^ statistics or Fisher’s exact test, respectively. All tests were two-tailed and the level of significance was set at 5% (*p* < 0.05). Participants were also stratified by sex and age (i.e., 18–39 and > 40 years). Age groups were set based on the increased prevalence of chronic diseases observed after 40 years of age in individuals with DS [20]. All analyses were performed using the IBM SPSS Statistics, version 25.0, software (IBM Corp., Armonk, NY).

## Results

We enrolled 64 adults with DS (mean age 36.4 years, SD 11.7, range 18–65), of whom 13 (20.3%) were women. From the initial NHANES population of 1498 individuals, 524 were excluded because of missing data for the variables of interest (n = 521) or height < 145 cm (n = 3). Following age and sex-matching, 256 individuals were included in the control group (mean age 36.3 years, SD 11.8, range 18–65), of whom 52 (20.3%) were women. The main anthropometric and body composition parameters of DS and NHANES participants, as a whole and according to sex, are listed in Tables 1 and 2, respectively.

**Table 1:** Parameters of body composition in participants with DS and NHANES controls

Variables	DS (n = 64)	NHANES (n = 256)	*p*-value
Age (mean, SD)	37.2 ± 11.5	36.3 ± 11.8	0.572
Women (n, %)	13 (20.3%)	52 (20.3%)	0.932
Height (cm)	156.1 ± 8.7	166.1 ± 9.0	** < 0.001**
Body mass (kg)	63.2 ± 13.5	77.5 ± 21.8	** < 0.001**
BMI (kg/m^2^)	25.9 ± 4.7	28.2 ± 7.0	**0.026**
4-m WS (m/s)	0.85 ± 0.33	0.95 ± 0.36	**0.001**
HG (kg)	18.7 ± 7.0	30.2 ± 11.5	** < 0.001**
HG/h2 (kg/m^2^)	7.5 ± 3.1	11.2 ± 4.5	** < 0.001**
Lean mass parameters			
Upper left limb (kg)	2.298 ± 0.624	2.627 ± 1.092	**0.022**
Upper right limb (kg)	2.558 ± 0.658	2.811 ± 1.134	**0.035**
Lower left limb (kg)	6.735 ± 1.248	7.414 ± 2.670	**0.049**
Lower right limb (kg)	6.778 ± 1.255	7.588 ± 2.695	**0.020**
Total body mass (kg)	43.762 ± 8.064	46.415 ± 15.481	**0.043**
ASM (kg)	18.370 ± 3.594	20.439 ± 7.446	** < 0.001**
SMI (kg/m^2^)	7.38 ± 1.01	7.46 ± 2.77	0.825
Fat mass parameters			
Total body fat (kg)	19.410 ± 7.958	24.315 ± 12.645	**0.003**
Percent fat mass (%)	29.43 ± 6.69	32.34 ± 8.24	**0.009**
Android fat mass (kg)	1.713 ± 0.883	2.103 ± 1.184	** < 0.001**
Gynoid fat mass (kg)	3.407 ± 1.335	3.978 ± 1.937	** < 0.001**
A/G ratio	0.98 ± 0.17	1.01 ± 0.22	0.115
FMI (kg/m^2^)	7.96 ± 3.18	8.92 ± 4.83	0.135

Values in bold are statistically significant at level < 0.05

BMI: body mass index; WS: walking speed; HG: dominant handgrip strength; ASM: appendicular skeletal muscle mass; SMI: skeletal muscle index; A/G ratio: android fat mass/gynoid fat mass ratio; FMI: fat mass index

**Table 2:** Parameters of body composition in DS participants and NHANES controls, according to sex (*NA* = not applicable)

Variables	Women			Men		
	NHANES (n = 52, 20.3%)	DS (n = 13, 20.3%)	*p*-value	NHANES (n = 204, 79.7%)	DS (n = 51, 79.7%)	*p*-value
Age (mean, SD)	37.5 ± 10.9	37.3 ± 11.2	**0.925**	37.7 ± 11.8	37.6 ± 11.8	0.935
Age range	18–57	23–56	NA	18–65	19–65	NA
Height (cm)	166.1 ± 9.9	152.8 ± 7.2	** < 0.001**	167.5 ± 9.4	156.7 ± 9.0	** < 0.001**
Weight (Kg)	92.9 ± 25.6	55.3 ± 8.5	** < 0.001**	91.7 ± 25.9	65.0 ± 13.9	** < 0.001**
BMI (kg/m^2^)	28.41 ± 8.24	26.41 ± 4.80	0.085	28.16 ± 6.92	23.72 ± 6.5	**0.004**
4 m ws(m/s)	0.92 ± 0.31	0.82 ± 0.32	**0.002**	0.96 ± 0.37	0.85 ± 0.33	** < 0.001**
HG (kg)	30.18 ± 11.96	16.93 ± 6.54	** < 0.001**	31.27 ± 11.95	17.26 ± 7.29	** < 0.001**
HG/h2 (kg/m^2^)	10.8 ± 4.5	8.2 ± 3.2	**0.004**	11.5 ± 4.5	7.1 ± 3.0	** < 0.001**
Lean mass parameters						
Upper left limb (kg)	2.491 ± 1.042	1.626 ± 0.615	** < 0.001**	2.621 ± 1.134	2.464 ± 0.557	0.332
Upper right limb (kg)	2.692 ± 1.121	1.829 ± 0.603	** < 0.001**	2.801 ± 1.171	2.715 ± 0.607	0.612
Lower left limb (kg)	7.019 ± 2.588	5.352 ± 0.869	**0.001**	7.377 ± 2.620	6.943 ± 1.213	0.467
Lower right limb (kg)	7.209 ± 2.638	5.647 ± 1.198	** < 0.001**	7.557 ± 2.664	6.936 ± 1.244	0.246
Total body mass (kg)	44.315 ± 15.161	36.795 ± 4.756	**0.003**	46.057 ± 15.067	47.007 ± 6.943	0.659
ASM (kg)	19.413 ± 7.246	14.455 ± 2.834	** < 0.001**	20.165 ± 7.366	19.059 ± 3.441	** < 0.001**
SMI (kg/m^2^)	7.05 ± 2.71	6.94 ± 1.19	0.978	7.34 ± 2.84	7.60 ± 0.93	0.167
Fat mass parameters						
Total body fat (kg)	22.832 ± 12.124	23.462 ± 7.777	0.764	23.505 ± 11.603	19.371 ± 8.420	**0.017**
Percent fat mass (%)	31.85 ± 8.24	38.12 ± 6.47	** < .001**	32.09 ± 8.35	28.32 ± 6.41	**0.003**
Android fat mass (kg)	2.121 ± 1.080	1.785 ± 0.851	**0.009**	2.125 ± 1.127	1.662 ± 0.910	** < 0.001**
Gynoid fat mass (kg)	4.888 ± 1.452	3.982 ± 1.228	** < 0.001**	3.345 ± 1.455	2.997 ± 1.264	** < 0.001**
A/G ratio	0.88 ± 0.20	0.89 ± 0.15	0.519	1.11 ± 0.22	1.04 ± 0.16	**0.002**
FMI (kg/m^2^)	8.11 ± 4.29	10.16 ± 4.35	** < 0.001**	8.61 ± 4.59	7.88 ± 3.33	0.286

Values in bold are statistically significant at level < 0.05

BMI: body mass index; WA: walking speed; HG: dominant handgrip strength; ASM: appendicular skeletal muscle mass; SMI: skeletal muscle index; A/G ratio: android fat mass/gynoid fat mass ratio; FMI: fat mass index

### Anthropometric data and body composition parameters

#### Anthropometric data

Both mass and height were lower in the DS sample compared with NHANES controls (63.2 ± 13.5 kg vs. 77.5 ± 21.8 kg, *p* < 0.001; 156.1 ± 8.7 cm vs. 166.1 ± 9.0 cm, *p* < 0.001). BMI was greater in controls than DS participants (28.2 ± 7.0 kg/m^2^ vs. 25.9 ± 4.7 kg/m^2^, *p* = 0.026). After sex stratification, BMI was similar between female participants from the two samples, whereas it was lower among DS men than in their NHANES peers (Table 2).

#### Fat mass parameters

In the NHANES sample, total fat mass (24.32 ± 12.65 kg vs. 19.41 ± 7.96 kg, *p* = 0.003), percent fat mass (32.8% ± 8.2% vs. 29.4% ± 6.7%, *p* = 0.009), android fat mass (2.10 ± 1.18 kg vs. 1.7 ± 0.88 kg, *p* < 0.001), and gynoid fat mass (3.98 ± 1.94 kg vs. 3.41 ± 1.34 kg, *p* < 0.001) were higher than in the DS sample. A/G and FMI were similar between the two samples. Men in the NHANES sample had greater total fat mass (23.51 ± 11.60 kg vs. 19.37 ± 8.42 kg, *p* = 0.017) and percent fat mass (32.1% ± 8.2% vs. 28.3% ± 6.4%, *p* = 0.001) compared with DS men. Percent fat mass was greater in DS women (38.1 ± 6.5% vs. 31.9 ± 8.2%, *p* < 0.001) compared with NHANES controls. The mean FMI was higher among DS women relative to their NHANES counterparts (10.16 ± 4.35 kg/m^2^ vs. 8.11 ± 4.29 kg/m^2^, *p* < 0.001). FMI was not significantly different between men with DS and NHANES peers (*p* = 0.286). Android and gynoid fat mass was significantly lower in DS individuals than in NHANES controls, both in the whole sample and in the two sexes separately (all *p* values < 0.001). The A/G ratio was not different between the two samples (*p* = 0.31). After sex stratification, mean A/G was significantly lower among men with DS than in NHANES controls (1.04 ± 0.16 vs. 1.11 ± 0.22, *p* = 0.002).

#### Lean mass parameters

As shown in Table 1, both total and segmental lean mass parameters were lower in DS than in the NHANES sample, as was ASM (all p values < 0.05). After stratification for sex, only women in the DS sample showed lower total and segmental lean mass parameters compared with their NHANES controls (all p values < 0.05). No significant differences were found in total or segmental lean mass between men with DS and their NHANES peers. SMI values were similar in DS and NHANES participants both in the whole sample (7.38 ± 1.01 kg/m^2^ vs. 7.46 ± 2.77 kg/m^2^, *p* = 0.825) and in the two sexes separately (Table 2).

### Muscle strength and physical performance

Handgrip strength was significantly lower in adults with DS compared with NHANES controls both in the whole sample (18.7 ± 7.0 kg vs. 30.2 ± 11.6 kg, *p* < 0.001) and in men and women separately (*p* < 0.001 for both). A similar pattern was observed after normalization of handgrip strength by height. Mean handgrip strength was above the EWGSOP2 cut-point for low muscle strength in women with DS (16.9 ± 6.5 kg), while it was below the cut-point for dynapenia in DS men (17.3 ± 7.3 kg). Finally, 4-m walking speed was slower in adults with DS compared with the NHANES sample (0.85 ± 0.33 m/s vs. 0.95 ± 0.36 m/s, *p* < 0.001).

### Sarcopenia and sarcopenic obesity

The frequency of sarcopenia and sarcopenic obesity in the two study samples is shown in Table 3. The frequency of sarcopenia was significantly higher in the DS than in the NHANES sample (n = 23, 35.6% vs. n = 51, 19.9%; *p* = 0.007), with sex-specific differences. Men with DS had more frequently sarcopenia than their NHANES counterparts (n = 20, 39.2% vs. n = 39, 19.1%; *p* = 0.002), while no differences were detected in women (n = 3, 23.1% vs. n = 12, 23.0%; *p* = 0.929). Sarcopenic obesity was diagnosed in four (6.3%) adults with DS and 33 (12.9%) NHANES participants (*p* = 0.137).

**Table 3:** Prevalence of sarcopenia and sarcopenic obesity in DS participants and NHANES sample controls

Variables	Whole samples (n = 320)			Men (n = 255)			Women (n = 65)		
	NHANES (n = 256)	DS (n = 64)	p-value	NHANES (n = 204)	DS (n = 51)	p-value	NHANES (n = 52)	DS (n = 13)	p-value
Sarcopenia	51 (19.9%)	23 (35.6%)	**0.007**	39 (19.1%)	20 (39.2%)	**0.002**	12 (23.0%)	3 (23.1%)	0.929
Sarcopenic obesity	33 (12.9%)	4 (6.3%)	0.137	25 (12.3%)	3 (5.8%)	0.802	8 (15.4%)	1 (7.7%)	0.674

Values in bold are statistically significant at level < 0.05

Table 4 shows the frequency of sarcopenia and sarcopenic obesity according to age groups. Among participants younger than 40, the frequency of sarcopenia was not significantly different between the DS (n = 11, 29.7%) and the NHANES sample (n = 39, 27.1%; *p* = 0.748). In those 40 years or older, sarcopenia was more frequent among DS participants than in their NHANES counterparts (n = 12, 44.4% vs. n = 12, 10.7%; *p* < 0.001). No significant age-specific differences were found in the frequency of sarcopenic obesity between the two samples.

**Table 4:** Prevalence of sarcopenia and sarcopenic obesity in DS participants and NHANES controls according to age groups

Variable	< 40 years (n = 181)									> = 40 years (n = 139)								
	Whole sample			Women (n = 40)			Men (n = 141)			Whole sample			Women (n = 25)			Men (n = 114)		
	DS (n = 37)	NHANES (n = 144)	P-value	DS (n = 8)	NHANES (n = 32)	*p*-value	DS (n = 19)	NHANES (n = 84)	*p*-value	DS (n = 27)	NHANES (n = 112)	*p*-value	DS (n = 5)	NHANES (n = 20)	*p*-value	DS (n = 22)	NHANES (n = 92)	*p*-value
Sarcopenia	11 (29.7%)	39 (27.1%)	0.748	1 (12.5%)	11 (34.4%)	0.396	10 (34.5%)	28 (25.0%)	0.305	12 (44.4%)	12 (10.7%)	< 0.001	2 (40.0%)	1 (5%)	0.090	10 (45.5%)	11 (12.0%)	** < 0.001**
Sarcobesity	2 (5.4%)	25 (17.4%)	0.075	1 (12.5%)	7 (21.9%)	0.553	1 (3.4%)	18 (16.1%)	0.123	2 (7.4%)	8 (7.1%)	0.962	0 (0.0%)	1 (5%)	0.610	2 (9.1%)	7 (7.6%)	0.817

Values in bold are statistically significant at level < 0.05

## Discussion

Findings from the present study indicate that body composition parameters and physical performance differ between adults with DS and matched controls enrolled in the NHANES, with age- and sex-specific patterns. The frequency of sarcopenia was found to be higher in individuals with DS 40 years and older compared with their NHANES controls. However, the frequency of sarcopenic obesity was not different between samples.

Although muscle hypotonia and increased adiposity are typical features of DS, evidence on differences in body composition and physical performance between adults with DS and the general adult population is sparse.

Adults with DS are typically shorter and lighter than the general population. However, whether and to what extent differences in body size translate into specific patterns in indexed parameters (e.g., BMI, SMI, FMI, A/G ratio) and body composition is unclear. In our study, BMI was lower in adults with DS compared with the NHANES sample. The mean BMI in individuals with DS was 25.9 kg/m^2^, indicating a condition of overweight, which is consistent with previous findings [21]. After sex stratification, BMI was lower in men with DS than in their NHANES peers, while it was not significantly different among female participants. A significantly smaller total and appendicular lean mass in comparison with NHANES controls was observed in women with DS. However, differences in appendicular lean mass were no longer evident after adjusting for body size.

Participants with DS also showed lower fat mass than NHANES controls. After sex stratification, percent fat mass and FMI were higher in women with DS compared with NHANES peers. An opposite pattern was observed in men. These findings are consistent with a previous study reporting a higher prevalence of obesity in women, but not in men with DS relative to matched controls [22]. The mechanisms underlying these sex-related differences are yet to determined, but seem to include metabolic [23], hormonal [24], and social and environmental factors. It is also noteworthy that, in our study, only men with DS showed a significantly lower A/G ratio than their NHANES counterparts. To the best of our knowledge, ours is the first work describing differences in A/G ratio between individuals with DS and the general population. Hormonal abnormalities (e.g., hypogonadism, hypothyroidism) might contribute to an altered fat metabolism and adipose tissue distribution [25].

Physical performance, as determined by 4-m walking speed and handgrip strength either absolute or normalized by height, was significantly lower in DS participants compared with the NHANES sample. The low handgrip strength values in adults with DS could be explained, at least partly, by a reduced ability to execute complex orders. Additional factors that may contribute to dynapenia in DS include lack of motivation to perform maximal strength testing [10], defects in nerve conduction velocity, changes in muscle fiber composition, abnormalities in myocyte mitochondria, and alterations of neuromuscular junctions [26]. Although handgrip strength has been proposed as a feasible and reliable test in adults with DS [19], arm length and hand span have been quantified and correlated with grip strength among children and adolescent with Down syndrome [27], and the possibility cannot be discarded that other muscle strength tests might be more suitable for these population. Sarcopenia was more frequent in the DS sample, which was mostly due dynapenia rather than a low SMI. After stratification for age, sarcopenia was more frequent only in adults with DS over 40 years of age, with a difference that appeared to be mainly driven by male participants. During “normal” aging, muscle strength declines earlier and faster than muscle mass [28]. Since musculoskeletal abnormalities are typical features of DS, our results could be due to a developmental defect of the muscle rather than accelerated aging. However, in a murine model of DS, adult mice show weakness and motor changes comparable to those observed in humans with DS [29]. Interestingly, these rodents display ultrastructural abnormalities in skeletal muscle fibers of the quadriceps femoris that occur earlier than in their wild-type littermates and resemble those observed in older adults with sarcopenia [5]. Altogether, these findings support the notion of DS as a progeroid condition also at the muscular level. Interestingly, adults with DS have lower bone mineral density (BMD) compared with the general population, and they experience a steeper decline in BMD with age [30]. Then, musculoskeletal abnormalities in DS could share similar aging patterns. Besides the genetic defect, additional factors that contribute to the loss of physical performance in DS include lower levels of physical activity [31] and unbalanced dietary regimens during adulthood [32]. However, neither of them was assessed in our study.

The observation that sarcopenia is frequent in adults with DS calls for the design of multicomponent interventions involving physical exercise and nutritional counseling to prevent the negative outcomes associated with premature muscle aging [33], as well as a comprehensive approach to manage the clinical complexity of adults with DS [34].

Finally, the frequency of sarcopenic obesity was not significantly different between adults with DS and the NHANES sample regardless of age. While data on fat mass and FMI suggest that sarcopenic obesity might be more frequent higher in adults with DS, especially in women, the small number of DS participants with sarcopenic obesity prevents from drawing meaningful conclusions about its actual prevalence in this population.

## Limitations

Although reporting novel findings, our study in not free of limitations. First, the sample size was relatively small. However, participants with DS were thoroughly characterized, which strengthens our findings. The presence of sarcopenia was established according to the EWGSOP2 criteria recommended for the general population. Specific cut-points for muscle mass and strength are needed to identify individuals with DS at risk of adverse events. The lack of information on nutrition and habitual physical activity did not allow for appreciating the possible influence of these factors on body composition and physical performance. Furthermore, since participants were relatively young, findings may not be generalized to older adults with DS, especially to older women with DS. Finally, data on controls were obtained from the 2001–2002 NHANES cycle and a cohort effect cannot be ruled out. In the control population, only data on some comorbid conditions were available. This prevented us from comparing clinical data in the two samples and conducting multivariate analyses with chronic diseases as covariates.

## Conclusions

Findings from the present study indicate that adults with DS have a peculiar body composition and fat distribution that differ from the NHANES population. These characteristics should be considered as part of the phenotypic manifestations of chromosome 21 trisomy and may contribute to increasing the risk of physical disability and other negative health-related events in individuals with DS. Overall, the results of the present study suggest that muscle mass and function should be assessed routinely in individuals with DS to timely identify those at risk of negative outcomes and in whom interventions should be prescribed to attenuate or reverse the loss of muscle mass and strength.

## Data Availability

To protect study participant privacy, data cannot be shared openly and are available on request from the corresponding author. NHANES data are openly available at https://www.cdc.gov/nchs/nhanes/index.htm.
